# Effects of bisoprolol and cilazapril on the central retinal artery blood flow in patients with essential hypertension—preliminary results

**DOI:** 10.3109/03009734.2010.487951

**Published:** 2010-10-27

**Authors:** Andrzej Madej, Stanisława Gierek-Ciaciura, Maciej Haberka, Joanna Lekston-Madej, Marcin Basiak, Olga Domańska, Bogusław Okopień

**Affiliations:** ^1^Department of Internal Medicine and Clinical Pharmacology, Medical University of Silesia, KatowicePoland; ^2^Department of Ophthalmology, Medical University of Silesia, KatowicePoland

**Keywords:** Bisoprolol, central retinal artery, cilazapril, Doppler ultrasonography

## Abstract

**Background:**

A growing body of evidence suggests that effective blood pressure reduction may inhibit the progression of microvascular damage in patients with essential arterial hypertension. However, the potential influence of anti-hypertensive drugs on ocular circulation has not been studied sufficiently.

**Purpose:**

The aim of our study was to evaluate the effects of anti-hypertensive therapy on blood flow in the central retinal artery in patients with systemic arterial hypertension.

**Material and methods:**

Twenty patients with essential arterial hypertension, aged 32–46 years, were examined with Doppler ultrasonography (10 MHz ultrasound probe). Blood flow velocities, pulsatility, and vascular resistance were determined before and 3 hours after systemic application of either bisoprolol 5 mg or cilazapril 2.5 mg.

**Results:**

Administered bisoprolol significantly decreased maximum (9.8 ± 0.5 cm/s versus 8.5 ± 0.6 cm/s; *P* < 0.05) and minimum (2.75 ± 0.19 cm/s versus 1.75 ± 0.27 cm/s; *P* < 0.02) velocity, increased the Pourcellot's index (0.71 to 0.79; *P* < 0.05) in central retinal artery. There were no statistically significant changes in central retinal artery blood flow after administration of cilazapril.

**Conclusion:**

Systemic application of beta-blockers may unfavourably disturb the ocular blood flow.

## Introduction

Systemic arterial hypertension gradually contributes to progressive damage of arterial vessels. Chronic increases in blood pressure cause several responses within the microvasculature, including endothelial dysfunction, impaired vessel relaxation, enhanced contractile response, and complex intima-media thickening. Finally, hypertrophy and proliferation of vascular smooth muscle cells decrease lumen diameter, which is accompanied by an increase in vessel resistance (1). It is well known that effective reduction of blood pressure may slow down progression of small arteries' damage. Recent guidelines for the management of arterial hypertension focus on the necessity of intensive blood pressure lowering. Clinical trials have clearly shown that several classes of drugs, including beta-blockers (BB) and angiotensin-converting enzyme inhibitors (ACEI), reduce the complications of systemic hypertension (2). Whereas beta-adrenergic receptors and components of tissue renin-angiotensin system (RAS) receptors have been localized within the ophthalmic circulation (3–6), potential unfavourable anti-hypertensive drug effects on ocular blood flow must be considered before pharmacotherapy selection. Among glaucoma vascular risk factors, special attention is paid to systemic arterial hypertension, hypotension, and nocturnal hypotension (‘big dippers’). Moreover, patients with hypertension reveal impaired physiological ocular blood flow auto-regulation with range shifted toward higher intra-ocular pressure (IOP) values, which sensitizes them to lower systemic blood pressure (7).

According to our knowledge, data concerning influence of different anti-hypertensive drugs applied systemically on ocular blood flow in humans are very scarce. Therefore the aim of our study was to evaluate the influence of BB (bisoprolol) and ACEI (cilazapril) on central retinal artery (CRA) flow assessed with Doppler ultrasonography in patients with systemic arterial hypertension.

## Patients and methods

We enrolled 20 patients (aged 32–46 years) with a newly diagnosed essential arterial hypertension requiring monotherapy with one of the anti-hypertensive drugs. Patients were divided into two study groups consisting of 10 patients each and were given orally once (one single dose) either cilazapril 2.5 mg (Inhibace; Roche, Warsaw, Poland) in group 1, or bisoprolol 5 mg (Concor; Merck, Warsaw, Poland) in group 2. The main exclusion criteria were: any ophthalmic disorders (including glaucoma) and any systemic diseases requiring vasoactive pharmacotherapy or affecting IOP. Additionally, the blood pressure before Doppler examination must have been lower than 140/90 mmHg. The evaluation of the CRA flow was carried out twice: before and 3 hours after taking the examined hypotensive drug. All Doppler measurements were performed in the vertical position. After an application of ultrasound gel, a 10 MHz probe (DRG Retina Doppler; Tomey, Cambridge, MA, USA) was positioned over the upper eyelid temporally to the optical axis of the eye, avoiding pressure to the eye-ball. Further analysis included: maximum (systolic) and minimum (diastolic) velocity, and Pourcellot's index of resistance (RI). Additionally, we examined the visual acuity, tonometry, and arterial blood pressure. The results were analysed with Wilcoxon's paired test. All text and table results are expressed as means ± SE. The study was performed in the Department of Ophthalmology, Medical University of Silesia in Katowice. The study was approved by the local ethics committee, and all patients gave written informed consent prior to enrolment.

## Results

There were no statistically significant differences in base-line values of blood pressure between the study groups. Administration of both study drugs resulted in a substantial systolic and diastolic blood pressure decrease (Table I). While a significant maximum and minimum blood velocity decrease and a Pourcellot's resistance index increase were observed in group 1 patients within 3 hours after bisoprolol 5 mg administration (Table II), in group 2 patients application of cilazapril 2.5 mg did not significantly affect the examined blood velocity parameters (Table III).

**Table I.** Blood pressure before and after administration of hypotensive drugs.

	SBP		DBP	
	Base-line	After drug administration	Base-line	After drug administration
Bisoprolol	134.3 ± 2.9	123.2 ± 3.1; *P* < 0.03	83.6 ± 1.7	75.1 ± 1.6; *P* < 0.05
Cilazapril	138.1 ± 2.6	125.1 ± 3.3; *P* < 0.05	84.9 ± 1.1	75.6 ± 1.9; *P* < 0.05

SBP = systolic blood pressure; DBP = diastolic blood pressure.

**Table II.** Doppler blood flow parameters in central retinal artery before and after bisoprolol administration.

	Base-line	After bisoprolol	Significance
V max (cm/s)	9.80 ± 0.5	8.50 ± 0.6	*P* < 0.05
V min (cm/s)	2.75 ± 0.19	1.75 ± 0.001	*P* < 0.02
RI	0.71 ± 0.01	0.79 ± 0.08	*P* < 0.05

V max = peak systolic velocity; V min = diastolic velocity; RI = resistance index.

**Table III.** Doppler blood flow parameters in central retinal artery before and after cilazapril administration.

	Base-line	After cilazapril	Significance
V max (cm/s)	8.90 ± 0.6	8.96 ± 0.4	ns
V min (cm/s)	2.10 ± 0.13	2.62 ± 0.01	ns
RI	0.77 ± 0.01	0.71 ± 0.05	ns

V max = peak systolic velocity; V min = diastolic velocity; RI = resistance index.

## Discussion

Our preliminary observations showed a different influence of BB and ACEI on the blood flow parameters within the CRA. Moreover, the obtained comparable values of blood pressure before and after both study drugs administration suggest that those effects are dependent on distinct mechanisms of action rather than blood pressure reduction degree. To the best of our knowledge, there are only a few small and inconsistent studies evaluating the influence on ocular circulation parameters of a short-term therapy or a single administration of an anti-hypertensive drug in patients with hypertension. Steigerwalt et al. and others observed an improvement in central retinal artery flow velocity in hypertensive patients after a 1-week treatment with trandolapril, but it did not reach flow parameters observed in healthy individuals (8). However, Kutschbach et al. found no changes in perifoveal intercapillary areas and mean perifoveal capillary velocities assessed in fluorescein angiograms in patients with hypertension treated with either BB or ACEI or calcium channel-blocker (CCB) (9). Among angiotensin II receptor blockers (ARBs), while candesartan monotherapy was shown to restore the proper vascular reactivity (10), valsartan increased blood flow velocity and decreased the resistance index of the CRA in essential hypertension (11), and it did not improve retinal endothelial function in elderly hypertensive patients (12). Moreover, Schocket et al. and others did not observe any substantial improvement in retinal blood flow in volunteers after oral administration of felodipine (13).

The above-described effects might be related to the different impact of anti-hypertensive drugs on microcirculation and endothelium function. Apart from the third-generation agents, studies on beta-blocker effects on endothelial function showed inconsistent results. The majority of studies demonstrated that ACEIs improve microcirculation endothelial function. ACEIs, especially agents with affinity to tissue RAS (cilazapril), not only efficiently inhibit angiotensin II influence on endothelium, but also enhance bradykinin-induced vasodilatation (14). In a double-blind randomized study, patients with essential hypertension treated with either atenolol or irbesartan showed comparable, well controlled blood pressure. However, gluteal subcutaneous biopsies revealed small arteries structure and function improvement only in individuals treated with irbesartan (15).

All major components of RAS have been identified within the retina of humans and rodents. However, the influence of systemically used ACEIs on CRA flow parameters has hardly been evaluated. The small and non-significant increase of velocity and decrease of vascular resistance suggest similar effects to systemic circulation.

Isolated human posterior ciliary arteries and monkeys' ophthalmic and ciliary arteries have been shown to contract mainly in response to noradrenaline and alpha-1 adrenoreceptor agonists with a lack of response to beta-agonists. However, using radioligand binding and auto-radiographic techniques Elena et al. and others localized beta-adrenoreceptors in the ocular circulation, particularly around the ciliary body and also in bovine retinal arteries (6,16,17). Acting via alpha-1 receptors, norepinephrine causes vasoconstriction of posterior ciliary and ophthalmic arteries. Bisoprolol-induced beta-receptor blockade may enhance alpha-1 receptor activation and finally increase the vascular resistance and decrease the flow velocity. Additionally, heart rate suppression caused by beta-blockers may intensify these effects (18,19).

The above results are based on a small study of a single administration of a hypotensive drug; they constitute preliminary observations and need to be verified in a larger population with a longer treatment period. Anyhow, the influence of different hypotensive drugs on ocular circulation parameters in hypertensive patients without glaucoma has not been evaluated sufficiently.

Our results suggest that ACEIs do not disturb or may slightly improve the blood flow in retinal vessels. Unfortunately, BBs may cause unfavourable changes in retinal microcirculation. Those additional effects might be very important in hypertensive patients with any pre-existing ocular disorders. Moreover, considering similarities between ocular and brain vessels, favourable effects on ocular circulation might also suggest improvements in brain circulation and contribute to stroke prevention.
